# Heritable Tissue-Culture-Free Gene Editing in *Nicotiana benthamiana* through Viral Delivery of SpCas9 and sgRNA

**DOI:** 10.1093/pcp/pcae100

**Published:** 2024-08-31

**Authors:** Tetsuya Yoshida, Masayuki Ishikawa, Seiichi Toki, Kazuhiro Ishibashi

**Affiliations:** Institute of Agrobiological Sciences, National Agriculture and Food Research Organization, 2-1-2 Kannondai, Tsukuba, Ibaraki 305-8518, Japan; Institute of Agrobiological Sciences, National Agriculture and Food Research Organization, 2-1-2 Kannondai, Tsukuba, Ibaraki 305-8518, Japan; Institute of Agrobiological Sciences, National Agriculture and Food Research Organization, 2-1-2 Kannondai, Tsukuba, Ibaraki 305-8518, Japan; Faculty of Agriculture, Ryukoku University, 1-5 Yokotani, Seta Oe-cho, Otsu, Shiga 520-2194, Japan; Graduate School of Nanobioscience, Yokohama City University, 22-2 Seto, Kanazawa-ku, Yokohama, Kanagawa 236-0027, Japan; Kihara Institute for Biological Research, Yokohama City University, 641-12 Maioka-cho, Totsuka-ku, Yokohama, Kanagawa 244-0813, Japan; Institute of Agrobiological Sciences, National Agriculture and Food Research Organization, 2-1-2 Kannondai, Tsukuba, Ibaraki 305-8518, Japan

**Keywords:** Gene editing, Nepovirus, Plant virus, Virus vector

## Abstract

Conventional plant gene editing requires laborious tissue-culture-mediated transformation, which restricts the range of applicable plant species. In this study, we developed a heritable and tissue-culture-free gene editing method in *Nicotiana benthamiana* using tobacco ringspot virus (TRSV) as a vector for in planta delivery of Cas9 and single-guide RNA (sgRNA) to shoot apical meristems. *Agrobacterium*-mediated inoculation of the TRSV vector induced systemic and heritable gene editing in *Nicotiana benthamiana PHYTOENE DESATURASE*. Transient downregulation of RNA silencing enhanced gene editing efficiency, resulting in an order of magnitude increase (0.8–13.2%) in the frequency of transgenerational gene editing. While the TRSV system had a preference for certain sgRNA sequences, co-inoculation of a TRSV vector carrying only Cas9 and a tobacco rattle virus vector carrying sgRNA successfully introduced systemic mutations with all five tested sgRNAs. Extensively gene-edited lateral shoots occasionally grew from plants inoculated with the virus vectors, the transgenerational gene editing frequency of which ranged up to 100%. This virus-mediated heritable gene editing method makes plant gene editing easy, requiring only the inoculation of non-transgenic plants with a virus vector(s) to obtain gene-edited individuals.

## Introduction

Gene editing in plants is a promising technology in agriculture that can confer beneficial traits, such as enhanced tolerance to biotic and abiotic stresses or increased nutrient values, to crops (Pixley et al. 2022). Plant gene editing requires the expression or introduction in cells of sequence-specific nucleases (SSNs) such as Cas9 and single-guide RNA (sgRNA) from the CRISPR–Cas9 system, followed by the regeneration of gene-edited plants via tissue culture. As tissue culture is time- and labor-intensive, often causes unwanted somatic mutations in the genome and is available for only a limited range of plant species or cultivars, it has been a major bottleneck in the application of gene editing to various plants (Altpeter et al. 2016).

Efforts have been made to circumvent tissue culture in plant gene editing (Nasti and Voytas 2021, Yang et al. 2023). Virus vectors are used widely for expression of foreign genes in planta, and adoption of their use in gene editing has been attempted (Uranga and Daròs 2023, Zhang et al. 2022). However, due to technical difficulties, including the large size of SSN-encoding genes (e.g. the *Cas9* gene from *Streptococcus pyogenes, SpCas9*, is about 4.1 kb in length) and the intrinsic antiviral activity of plant shoot apical meristems (Bradamante et al. 2021), entirely virus vector–mediated tissue-culture-free gene editing has proved to be unsuccessful so far. The meristem barrier of virus invasion can be partially overcome by inoculation of several virus vectors carrying sgRNA that may harbor functional sequences to facilitate the mobility of RNA in plants to Cas9-expressing transgenic plants (Ellison et al. 2020, Lei et al. 2021, Li et al. 2021, Uranga et al. 2021, Nagalakshmi et al. 2022). However, expression of Cas9 from plant virus vectors remains difficult and targeted mutagenesis has been successful in only a limited number of cases in somatic cells but has not been possible in germline cells (Ariga et al. 2020, Ma et al. 2020, Liu et al. 2023, Lee et al. 2024).

In this study, we aimed to develop a virus vector with the combined ability to invade plant meristems and express Cas9 and sgRNA to induce heritable gene editing in non-transgenic plants. This virus vector could eliminate the requirement for tissue culture in plant gene editing.

## Results and Discussion

**Fig. 1:**
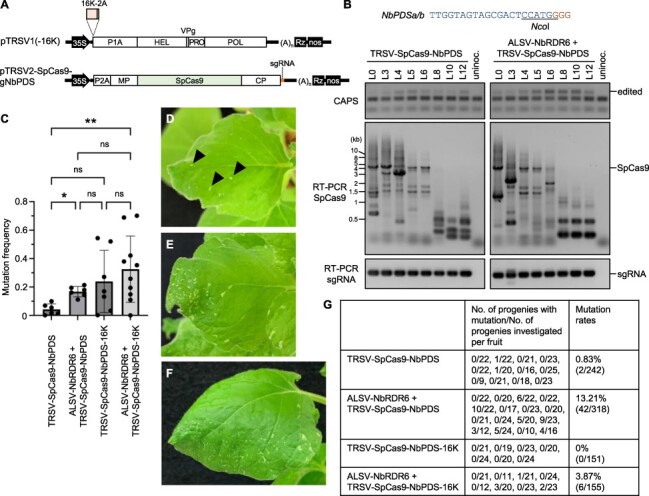
TRSV vector–mediated heritable gene editing in *N. benthamiana*. (A) Schematic representation of TRSV vectors. 35S, CaMV 35S promoter; (A)*_n_*, poly(A) sequence; Rz, hepatitis delta virus ribozyme; nos, *nopaline synthase* terminator; 16K, RNA silencing suppressor derived from TRV; 2A, porcine teschovirus-1 2A sequence; P1A, HEL, VPg, PRO, POL, P2A, MP and CP are TRSV-encoded proteins. (B) Systemic gene editing in TRSV vector–inoculated *N. benthamiana* plants. The 20-nt sgRNA target sequence and the GGG PAM sequence are shown in blue and brown, respectively, and the *Nco*I restriction site is underlined. (Upper) CAPS analysis of the *NbPDS* gene in inoculated (L0), third to sixth (L3–L6), eighth (L8), 10th (L10) and 12th (L12) upper leaves from an inoculated leaf. The positions of undigested DNA bands representing successful gene editing in the target site are indicated as ‘edited’. (Middle and lower) RT-PCR analyses for the stability of *SpCas9* (middle) and sgRNA (lower) sequences in the TRSV vector. ‘Uninoc’ indicates an uninoculated negative control plant. (C) Amplicon sequencing results for mutation frequency of the target site in the 10th upper leaves of plants inoculated with the indicated virus vectors. Bar graphs and error bars represent the mean values and standard deviations, respectively. Values of each plant are plotted in the graph and shown in Fig. S1D, E and S2D, E in detail. Statistical significance was determined using Brown-Forsythe and Welch ANOVA tests with Dunnett T3 multiple comparison test. ns, * and ** indicate *P* > 5.00 × 10^−2^, *P* = 1.01 × 10^−3^ and 2.06 × 10^−2^, respectively. Sample numbers are 6, 6, 7 and 10 for TRSV-SpCas9-NbPDS, ALSV-NbRDR6 + TRSV-SpCas9-NbPDS, TRSV-SpCas9-NbPDS-16K and ALSV-NbRDR6 + TRSV-SpCas9-NbPDS-16K, respectively. (D–F), An example of partially photobleached upper uninoculated leaf of plants inoculated with ALSV-NbRDR6 and TRSV-SpCas9-NbPDS (D), TRSV-SpCas9-NbPDS-16K (E) or ALSV-NbRDR6 and TRSV-SpCas9-NbPDS-16K (F). Arrowheads in (D) indicate photobleached spots. (G) Heritability of the introduced mutations. Mutation frequencies of each allele and nucleotide sequences of the target sites with mutations are shown in **Supplementary Table S1 and S2**, respectively.

As a suitable vector for tissue-culture-free gene editing, we selected tobacco ringspot virus (TRSV) because, unlike many other plant viruses, it can invade meristematic tissues of tobacco plants (Dong et al. 2010). TRSV belongs to the genus *Nepovirus* and has two-segmented positive-sense RNA genomes, each of which encodes a large polyprotein that is cleaved into mature proteins. As RNA1 (∼7.5 kb) and RNA2 (∼3.9 kb) are packaged separately in icosahedral capsids (Chandrasekar and Johnson 1998), we anticipated that TRSV RNA2 has extra coding capacity of at least 3.6 kb from the size difference between RNA1 and RNA2. We constructed cDNA clones of RNA1 and RNA2 of TRSV. The *SpCas9* gene was inserted in frame with the polyprotein of RNA2, and the sgRNA sequence targeting the *Nicotiana benthamiana PHYTOENE DESATURASE (NbPDS)* gene encoding an enzyme involved in carotenoid biosynthesis was inserted in the 3′ untranslated region of RNA2 (**Fig. 1A**). The resulting plasmid, designated as pTRSV2-SpCas9-gNbPDS, and the RNA1-encoding plasmid pTRSV1, collectively referred to here as TRSV-SpCas9-NbPDS, were introduced into *Agrobacterium* and inoculated into *N. benthamiana* via leaf infiltration. The sgRNA fragment of ∼0.1-kb long was stably maintained in the TRSV vector throughout the infected plants, while the *SpCas9* gene of ∼4.1-kb long was maintained several leaves distant from the inoculated leaves but deleted in higher leaves (**Fig. 1B**). Even in upper leaves, in which the infecting TRSV vector lost the *SpCas9* gene, mutations were detected in the target site of *NbPDS* (**Fig. 1B**). These results suggest that TRSV-SpCas9-NbPDS invaded shoot apical meristems before losing the SpCas9 sequence, induced gene editing in meristematic tissues and then lost the SpCas9 sequence during its spread into newly developed, gene-edited tissues derived from the edited meristems.

To improve the efficiency of TRSV-mediated gene editing, the *N. benthamiana RNA-DEPENDENT RNA POLYMERASE6 (NbRDR6)* gene—a key player in the plant antiviral RNA silencing system (Qu et al. 2005, Schwach et al. 2005)—was downregulated by virus-induced gene silencing (VIGS) using another virus vector derived from apple latent spherical virus (ALSV) that induces efficient VIGS in *N. benthamiana* and other plants (Igarashi et al. 2009) (**Supplementary Fig. S1A**). *NbRDR6*-VIGS plants showed higher gene editing efficiency than non-VIGS plants, with more severe symptoms of TRSV (e.g. mosaic and distortion of leaves) after TRSV-SpCas9-NbPDS inoculation (**Fig. 1B, C**; **Supplementary Fig. S1B–F**). In ALSV-NbRDR6- and TRSV-SpCas9-NbPDS-co-inoculated plants, gene-edited leaves developed sporadically but continuously (**Supplementary Fig. S1G**) and small photobleached spots, suggestive of a tetra-allelic knockout of *NbPDS* in the amphidiploid plant *N. benthamiana*, were occasionally found (**Fig. 1D**). Thus, suppression of RNA silencing could enhance TRSV-mediated gene editing, producing a mosaic pattern of gene-edited and non-edited cells. No photobleaching or mutation in the *NbPDS* target site was detected in upper leaves of plants inoculated with ALSV-NbRDR6 and TRSV-SpCas9, which lacks a sgRNA sequence (**Supplementary Fig. S1H**).

Given the improvement in efficiency of TRSV-mediated gene editing by attenuation of RNA silencing, we modified the TRSV vector to additionally encode 16K—the RNA silencing suppressor of tobacco rattle virus (TRV), another atypical positive-sense RNA virus that can invade meristematic tissues (Martín-Hernández and Baulcombe 2008)—in RNA1 to construct a virus vector that shows higher gene editing efficiency (**Fig. 1A**). Upon inoculation of the resulting vector (TRSV-SpCas9-NbPDS-16K) into *N. benthamiana* plants, TRSV symptoms appeared at a lower frequency than TRSV-SpCas9-NbPDS for unknown reasons (**Supplementary Fig. S2A, B**). Although the gene editing efficiencies induced by TRSV-SpCas9-NbPDS-16K were not statistically significant from those induced by TRSV-SpCas9-NbPDS because of the large variation, highly gene-edited individuals were often found (**Fig. 1C** and **Supplementary Fig. S2C–E**). TRSV-SpCas9-NbPDS-16K induced more intensively photobleached leaves than TRSV-SpCas9-NbPDS (**Fig. 1E, F**).

We then examined the heritability of mutations introduced by the TRSV vector. We screened plants presumably with gene-edited meristems by cleaved amplified polymorphic sequence (CAPS) in the 10th upper leaves from TRSV-inoculated leaves where the SpCas9 sequence is deleted from the TRSV genome in most cases and collected the first mature fruit of the plants. While TRSV infection impaired the fertility of *N. benthamiana*, we found gene-edited progenies from TRSV-SpCas9-NbPDS-inoculated plants with low frequency (2/242; 0.8%) but not from TRSV-SpCas9-NbPDS-16K-inoculated plants (0/151) (**Fig. 1G** and **Supplementary Table S1**). Pre-inoculation of ALSV-NbRDR6 enhanced the frequency of transmission of the targeted mutations to the next generation (42/318; 13.2% for TRSV-SpCas9-NbPDS and 6/155; 3.9% for TRSV-SpCas9-NbPDS-16K) (**Fig. 1G** and **Supplementary Table S1**). We currently have no explanation to account for the lower frequency of heritable gene editing by TRSV-SpCas9-NbPDS-16K than TRSV-SpCas9-NbPDS. Sanger sequencing confirmed that the edited progeny (E1) plants had mutations in *NbPDSa* and/or *NbPDSb* (**Supplementary Table S2**). An introduced mutation was inherited to the further (E2) generation following a Mendelian manner (**Supplementary Fig. S3**).

**Fig. 2:**
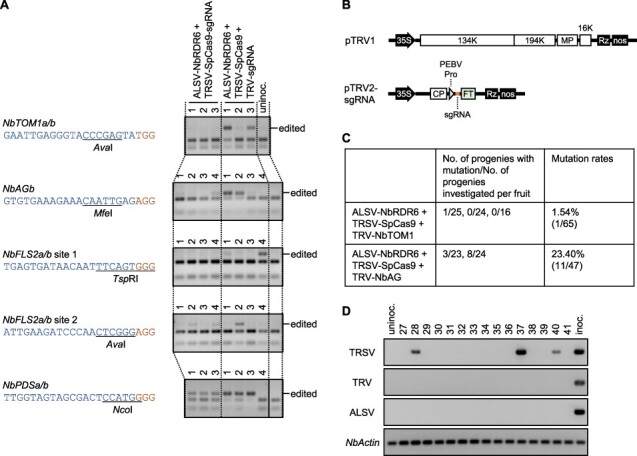
Gene editing by co-inoculation of TRSV-SpCas9 and TRV carrying sgRNA. (A) Detection of mutations in the *NbTOM1a/b, NbAGb, NbFLS2a/b* target site 1 or 2 and *NbPDSa/b* by CAPS analysis in the 10th upper leaves from TRSV (and TRV)-inoculated leaves of *N. benthamiana* plants inoculated with virus vectors denoted. The 20-nt sgRNA target sequence and the NGG PAM sequence are shown in blue and brown, respectively, and the recognition sites of restriction enzymes (*Ava*I, *Mfe*I, *Tsp*RI, *Ava*I and *Nco*I) are underlined. Lanes represent individual plants. The positions of undigested bands are indicated as ‘edited’. Lanes of the fifth panel were rearranged. (B) Schematic representation of TRV vectors. 35S, CaMV 35S promoter; Rz, self-cleaving ribozyme; nos, *nopaline synthase* terminator; PEBV Pro, subgenomic RNA promoter of pea early-browning virus; FT, full-length *Arabidopsis thaliana Flowering locus T* open reading frame sequence; 134K, 194K, MP, 16K and CP are TRV-encoded proteins. (C) Heritability of the introduced mutations in the *NbTOM1* or *NbAG* genes. Mutation frequencies of each allele and nucleotide sequences of the target sites with mutations are shown in **Supplementary Table S3 and S4**, respectively. (D) Detection of TRSV, TRV and ALSV RNAs in the progenies produced by seeds from plants inoculated with ALSV-NbRDR6, TRSV-SpCas9 and TRV-NbTOM1 by RT-PCR. The endogenous *N. benthamiana Actin* gene was used as an internal control. Progeny number 36 had the mutation. ‘Uninoc’ and ‘inoc’ indicate an uninoculated (negative control) and inoculated (positive control) *N. benthamiana* plant, respectively.

Other sgRNAs targeting the endogenous *N. benthamiana TOBAMOVIRUS MULTIPLICATION1* (*NbTOM1*)*, AGAMOUS* (*NbAG*) and *FLAGELLIN SENSING2* (*NbFLS2*) genes were tested for TRSV-based gene editing. Targeted mutations were found in the 10th upper leaves from TRSV-SpCas9-NbAG- or TRSV-SpCas9-NbFLS2-2-inoculated leaves of *NbRDR6*-VIGS *N. benthamiana* plants; however, mutations were hardly detectable in TRSV-SpCas9-NbTOM1- or TRSV-SpCas9-NbFLS2-1-inoculated plants (**Fig. 2A**). In TRSV vector–infected cells, sgRNA shortage for SpCas9 protein might be a problem as SpCas9 proteins are assumed to be produced by multiple rounds of translation from a single TRSV vector genomic RNA, while the genomic RNA itself is a source of sgRNA. To increase the supply of sgRNA, we co-inoculated a TRV vector carrying sgRNA (Ellison et al. 2020) (**Fig. 2B**) with TRSV-SpCas9. Plants co-inoculated with TRSV-SpCas9 and TRV vectors showed more severe disease symptoms than TRSV-SpCas9-sgRNA-inoculated plants, and the symptoms were further intensified by knockdown of *NbRDR6* (**Supplementary Fig. S4A–C**). Targeted mutations were successfully introduced by all five guide RNAs tested (NbTOM1, NbAG, NbFLS2-1, NbFLS2-2 and NbPDS) by co-inoculation of TRSV-SpCas9 and TRV-sgRNA (**Fig. 2A**). When TRV vector carrying sgRNA for *NbPDS* was co-inoculated with TRSV-SpCas9, intensively photobleached leaves were found (**Supplementary Fig. S4D, E**). The gene editing efficiency was reinforced by prior inoculation of ALSV-NbRDR6 (**Supplementary Fig. S4F, G**). These results suggest that the TRSV system has a relatively narrow sgRNA sequence preference, but the problem can be overcome by co-inoculation of a TRV vector carrying sgRNA. Although TRSV- and TRV-co-inoculated plants rarely bore seeds by themselves due to the severe symptoms, especially when *NbRDR6* expression was knocked down, we obtained gene-edited seeds from ALSV-NbRDR6-, TRSV-SpCas9- and TRV-NbTOM1- or TRV-NbAG-co-inoculated plants by cross-pollination of fertile pollens (**Fig. 2C** and **Supplementary Table S3, S4**). While TRSV, TRV and ALSV are seed-transmissible viruses (Yang and Hamilton 1974, Dikova 2005, Kamada et al. 2018), viral RNAs were detected in 3, 0 and 0, respectively, among 15 progeny plants by RT-PCR and the rest were free from detectable vertical transmission of the viruses (**Fig. 2D**).

**Fig. 3:**
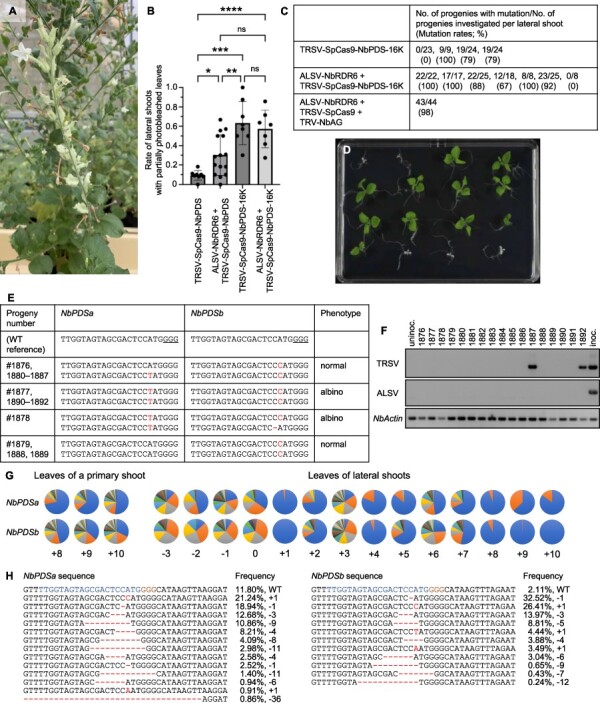
Gene editing in lateral shoots of TRSV vector–inoculated plants. (A) A near completely photobleached lateral shoot of a plant inoculated with TRSV-SpCas9-NbPDS. (B) The rate of lateral shoots bearing partially photobleached leaves. Bar graphs and error bars represent the mean values and standard deviations, respectively. Statistical significance was determined using Brown-Forsythe and Welch ANOVA tests with Dunnett T3 multiple comparison test. ns, *, **, *** and **** indicate *P* > 5.00 × 10^−2^, *P* = 1.10 × 10^−2^, 2.14 × 10^−2^, 8.60 × 10^−4^ and 2.03 × 10^−3^, respectively. Sample numbers are 8, 15, 8 and 7 for TRSV-SpCas9-NbPDS, ALSV-NbRDR6 + TRSV-SpCas9-NbPDS, TRSV-SpCas9-NbPDS-16K and ALSV-NbRDR6 + TRSV-SpCas9-NbPDS-16K, respectively. (C) Heritability of the introduced mutations for progenies from lateral shoots containing highly gene-edited tissues. Mutation frequencies of each allele and nucleotide sequences of the target sites with mutations are shown in **Supplementary Table S5–S8**. Phenotypes (D) and genotypes (E) of the progenies derived from a fruit harvested from a lateral shoot with highly photobleached leaves of a plant inoculated with ALSV-NbRDR6 and TRSV-SpCas9-NbPDS-16K with possible seed transmission of the virus (F). The PAM sequence is underlined, and a red letter and hyphen indicate a nucleotide insertion and deletion, respectively, in (E). TRSV (upper) and ALSV (middle) RNAs in the progenies were detected by RT-PCR in (F). Lane numbers correspond to the progeny numbers in (E). The endogenous *N. benthamiana Actin* gene (lower) was used as an internal control. ‘Uninoc’ and ‘inoc’ indicate an uninoculated (negative control) and inoculated (positive control) *N. benthamiana* plant, respectively. (G) Amplicon sequencing analysis of *NbPDSa* and *NbPDSb* in leaves from the primary and lateral shoots of a plant inoculated with ALSV-NbRDR6 and TRSV-SpCas9-NbPDS-16K. The unedited sequences are shown first in blue, followed by edited sequences in other colors in the order of read counts. Numbers indicate the position of nodes where the lateral branches or leaves arose. Zero, minus and plus indicate the node of an inoculated leaf, lower nodes and upper nodes, respectively. A biological replicate is shown in **Supplementary Fig. S6C**. (H) Nucleotide sequences around the target sites of *NbPDSa* and *NbPDSb* in a leaf from a lateral shoot that arose from the third lower node from the TRSV-inoculated leaf of the plant inoculated with ALSV-NbRDR6 and TRSV-SpCas9-NbPDS-16K [‘−3’ in (G)]. The 20-nt sgRNA target sequence and the GGG PAM sequence are shown in blue and brown, respectively. A red letter and hyphen indicate a nucleotide insertion and deletion, respectively. Numbers of nucleotide insertion (+) and deletion (−) are denoted. WT indicates the sequence with no mutations.

Mutations in lateral shoots called ‘sports’ are utilized in isolating mutants, especially in plants that propagate vegetatively (Foster and Aranzana 2018). We occasionally found extensively photobleached leaves or near completely whitened shoots resembling bud sports in lateral shoots of TRSV-SpCas9-NbPDS-inoculated plants (**Fig. 3A**). Then, we removed the apical buds of plants that have the gene-edited 10th upper leaves from TRSV-inoculated leaves to induce growth of lateral shoots and investigated the gene editing efficiency. The expression of the RNA silencing suppressor 16K, while having little effect on the heritable gene editing from primary shoots (**Fig. 1G**), increased the frequencies of branches with partially photobleached leaves more than the knockdown of *NbRDR6* (**Fig. 3B**). Although the white shoots did not bear seeds, we harvested fruits from lateral shoots with highly photobleached leaves of TRSV-SpCas9-NbPDS-16K-inoculated plants. A large proportion or all of progenies from most of such fruits were gene-edited (**Fig. 3C** and **Supplementary Table S5, S6**). The progenies included albino plants that had tetra-allelic mutations in *NbPDS* (**Fig. 3D, E**) that were not found in progenies from the primary shoot-derived seeds (**Supplementary Table S1, S2**). The seed transmission rate of TRSV and ALSV was 5.6% (2/36) and 0% (0/36), respectively, under our conditions, and we could obtain gene-edited individuals including albino plants without detectable viral RNA accumulation (e.g. #1876–1886 and #1888–1891 in **Fig. 3F**). Amplicon sequencing found lateral shoot tissues with reduced diversity of introduced mutations compared with the primary shoot tissues, suggesting that a genetic bottleneck can occur in lateral buds, which facilitated isolation of highly gene-edited shoots (**Fig. 3G, H**; **Supplementary Fig. S5, S6**). Highly gene-edited lateral shoots screened by CAPS in ALSV-NbRDR6-, TRSV-SpCas9- and TRV-NbAG-co-inoculated plants produced gene-edited progenies at 97.7% (43/44) after cross-pollination of fertile pollens (**Fig. 3C** and **Supplementary Table S7, S8**). Thus, we suggest that growing and screening lateral shoots from virus vector–inoculated plants is an easy and efficient way to isolate gene-edited plants by either seed or cutting propagation (**Supplementary Fig. S7**).

Now that we are 10 years into the CRISPR–Cas9 era, gene editing has become a standard method for exploring gene functions in model plants and improving traits in several crops. However, it is still difficult or impractical for many plants, due mostly to the inability to perform genetic transformation through tissue culture. Thus, the virus vector-based germline editing system demonstrated here has the potential to greatly expand the application of gene editing in plants. In addition, the simplicity and ease of this system could make it a standard method of gene editing for many plants. A technical limitation of virus-mediated gene editing is the host range of the virus vector, and the need for multiple virus vectors for efficient gene editing further limits the applicability of this system. TRSV has a wide host range and infects hundreds of plant species, including *Solanaceae, Cucurbitaceae*, *Fabaceae* and *Rosaceae* plants, and may be applicable to germline editing of these plants. For non-host plants for TRSV, other viruses in the genus *Nepovirus* with diverse host ranges (Fuchs et al. 2017) may also be used for delivery of Cas9 into meristems. ALSV and TRV also have broad host ranges (Igarashi et al. 2009, Shi et al. 2021), and many other viruses demonstrated for their applications in VIGS and sgRNA expression vector could be used in combination with a Cas9-expressing nepovirus vector.

## Materials and Methods

### Plasmids

Synthetic full-length cDNAs of TRSV RNA1 and RNA2 with a 3′-terminal hepatitis delta virus ribozyme sequence were cloned between the cauliflower mosaic virus (CaMV) 35S promoter and *nopaline synthase* terminator in pPZP2028 (Endo et al. 2015) to construct pTRSV1 and pTRSV2-MCS, respectively. The TRV 16K-coding sequence fused with a porcine teschovirus-1 2A sequence (Kim et al. 2011) was inserted to pTRSV1 to construct pTRSV1-16K. The SpCas9-coding sequence optimized for expression in plants (Fauser et al. 2014) and sgRNA sequences comprising an 80-bp scaffold and guide RNAs for *NbPDS* (5′-TTGGTAGTAGCGACTCCATG-3′), *NbTOM1* (5′-GAATTGAGGGTACCCGAGTA-3′), *NbAGb* (5′-GTGTGAAAGAAACAATTGAG-3′), *NbFLS2* target site 1 (5′-TGAGTGATAACAATTTCAGT-3′) or *NbFLS2* target site 2 (5′-ATTGAAGATCCCAACTCGGG-3′) were inserted into pTRSV2-MCS to construct pTRSV2-SpCas9-gNbPDS, pTRSV2-SpCas9-gNbTOM1, pTRSV2-SpCas9-gNbAG, pTRSV2-SpCas9-gNbFLS2-1 or pTRSV2-SpCas9-gNbFLS2-2, respectively. Nucleotide sequences of pTRSV1, pTRSV1-16K and pTRSV2-SpCas9-gNbPDS were deposited in DNA Data Bank of Japan (DDBJ) under accession numbers LC764403, LC764404 and LC764405, respectively.

ALSV cDNA-containing plasmids (Li et al. 2004, Igarashi et al. 2009) were generous gifts from Dr. Nobuyuki Yoshikawa (Iwate University, Iwate, Japan). cDNAs for ALSV RNA1 and RNA2 were cloned between CaMV 35S promoter and *nopaline synthase* terminator in pPZP2028 (Endo et al. 2015) to generate pPZP-ALS1 and pPZP-ALS2m for agroinoculation. The 30-bp sequence encoding a protease processing site in pPZP-ALS2m immediately before the multi cloning site (*Xho*I-*Sma*I-*Bam*HI) was replaced by 5′-TTATTGGAGGGACAAGGTCCAGACTTTACT-3′ that has synonymous substitutions. To downregulate *NbRDR6* expression, a 201-bp partial fragment of *NbRDR6* (Niben101Scf12609g01010.1) was amplified using primers NbRDR6-XhoI-F and NbRDR6-BamHI-R (sequences of all primers used are presented in **Supplementary Table S9**) and inserted between *Xho*I and *Bam*HI sites of pPZP-ALS2m.

TRV cDNA-containing plasmids (Liu et al. 2002) for agroinoculation were generous gifts from Dr. Savithramma P. Dinesh-Kumar (University of California, Davis, CA). Pea early-browning virus subgenomic promotor and full-length *Arabidopsis thaliana FT* open reading frame sequence were inserted to the TRV RNA2-encoding plasmid according to a previous report (Ellison et al. 2020). The same guide RNA sequences as in TRSV vectors were inserted in TRV RNA2 vectors.

### Plant materials

*Nicotiana benthamiana* plants were grown on soil in a growth chamber (25°C, 16-h light/8-h dark) or a greenhouse (25°C, natural light conditions). ALSV vector was inoculated at about 2–3 weeks after germination. TRSV vector was challenge inoculated at about 1–3 weeks after ALSV inoculation. TRV vector was inoculated simultaneously with TRSV when needed. Inoculated plants whose 10th upper leaves from TRSV vector–inoculated leaves were positive for gene editing were selected for further analyses. To grow lateral shoots, the primary shoots were cut above the 10th upper leaves from TRSV-inoculated leaves, except for the analysis of *NbAGb* editing. Spontaneously grown lateral shoots were analyzed for *NbAGb*. For **Fig. 3B**, the number of secondary branches bearing leaves with partially photobleached areas among 10 leaves from a node was counted. Branches bearing fewer than 10 leaves were excluded from the analysis. For the analyses of the progeny plants, surface-sterilized seeds were sown on half-strength Murashige and Skoog medium with 0.5% (w/v) agar. Cross-pollination was carried out using fertile pollens from non-inoculated or ALSV-NbRDR6-inoculated *N. benthamiana* plants, followed by CAPS of calyces to select fruits for further analyses. Fruits from ALSV-NbRDR6-, TRSV-SpCas9- and TRV-NbTOM1- or TRV-NbAG-co-inoculated plants were selected.

### Virus inoculation

Virus inoculation was conducted via agroinfiltration essentially as described previously (Kaya et al. 2017). *Agrobacterium* strains C58C1 or GV2260 harboring the plasmid encoding the RNA silencing suppressor p19 of tomato bushy stunt virus and harboring viral infectious clones were mixed and infiltrated at final optical density at 600 nm of 0.2 and 0.5, respectively.

### Analysis of gene editing frequency and genotyping

For detection of possible gene editing in meristems, gene editing efficiency was examined by CAPS using DNA extracted from the 10th upper leaves from TRSV-inoculated leaves unless otherwise noted. DNA was extracted using Kaneka Easy DNA Extraction Kit version 2 (Kaneka, Tokyo, Japan) and subjected to PCR using primers NbPDS-CAPS-F1 and NbPDS-CAPS-R1 (for the *NbPDSa* and *NbPDSb* genes), NbTOM1s_CAPS_F and NbTOM1s_CAPS_R (for the *NbTOM1a* and *NbTOM1b* genes), NbAGgR1_B-CAPS-F1 and NbAGgR1_B-CAPS-R1 (for the *NbAGb* gene), NbFLS2-gR1CAPS-abF and NbFLS2-gR1CAPS-abR (for target site 1 of *NbFLS2a* and *NbFLS2b* genes), or NbFLS2-gR2CAPS-abF and NbFLS2-gR2CAPS-abR (for target site 2 of *NbFLS2a* and *NbFLS2b* genes) followed by digestion with *Nco*I, *Ava*I, *Mfe*I, *Tsp*RI or *Ava*I, respectively.

Genotyping of progeny plants was performed by fragment analysis or CAPS analysis and Sanger sequence as necessary. For fragment analysis of *NbPDS*, DNA extracted from true leaves was subjected to PCR using primers [HEX]NbPDSfw and NbPDSab-FR115rv. PCR products were analyzed using the SeqStudio Genetic Analyzer (Thermo Fisher Scientific, Waltham, MA).

For Sanger sequencing of the introduced mutations, each allele was amplified separately using primers NbPDS_A-CAPS-F1 and NbPDS_A-CAPS-R1 (for *NbPDSa*), NbPDS_B-CAPS-F1 and NbPDS_B-CAPS-R1 (for *NbPDSb*), NbTOM1_A-CAPS-F1 and NbTOM1_A-CAPS-R1 (for *NbTOM1a*), NbTOM1_B-CAPS-F1 and NbTOM1_B-CAPS-R1 (for *NbTOM1b*), and NbAGgR1_B-CAPS-F1 and NbAGgR1_B-CAPS-R1 (for *NbAGb*). PCR products were sequenced directly.

To carry out amplicon sequencing analysis of *NbPDSa* and *NbPDSb*, extracted DNA was subjected to first PCR using primers NbPDS-CAPS-F1 and NbPDS-CAPS-R1 followed by second PCR using primers NbPDS-AS-F3 and NbPDS-AS-R2. The third PCR was carried out using primers 3rdF and 3rdR, which have index sequences to distinguish amplicons, and the PCR products were subjected to sequencing analysis using Illumina MiSeq by Bioengineering Lab. Co., Ltd. (Kanagawa, Japan). Sequence data were analyzed using CRISPResso2 (Clement et al. 2019). To exclude sequencing errors, unique sequences less than 0.2% of total reads or with base substitutions around the target site (upstream 3 nts, 20-nt sgRNA target site and downstream 17 nts) were not counted. Original sequence data are available in DDBJ under accession numbers PRJDB17821 and PRJDB17822.

### RT-PCR analysis

Total RNA was extracted using RNAiso Plus (Takara, Shiga, Japan). RT-PCR was carried out using PrimeScript One Step RT-PCR Kit Ver.2 (Dye Plus) (Takara). For stability analyses of the SpCas9 or sgRNA sequences in a TRSV vector, primers TRSV2-1791F and TRSV2-1988R or TRSV2-3330F and TRSV2-3524R, respectively, were used. For seed transmissibility analyses, primers TRSV2-2725F and TRSV2-3046R, ALSV2-2241F and ALSV2-2554R or TRV2-rt-F1 and TRV2-rt-R1 were used to detect TRSV, ALSV or TRV RNA, respectively. Endogenous *NbActin* transcripts were detected using primers (Ma et al. 2020) NbActin-rt-F and NbActin-rt-R as an internal control.

To analyze the expression level of the *NbRDR6* gene, total RNA isolation and DNase treatment were carried out using ISOSPIN Plant RNA (Nippon Gene, Tokyo, Japan). cDNA was generated using the iScript gDNA Clear cDNA Synthesis Kit (Bio-Rad, Hercules, CA). Quantitative PCR was performed using the iQ5 Multicolor Real-Time PCR Detection System (Bio-Rad) with SsoAdvanced Universal SYBR Green Supermix (Bio-Rad). *NbPP2A* was amplified using primers (Liu et al. 2012) NbPP2A-rt-F and NbPP2A-rt-R as an internal control. *NbRDR6* was amplified using primers NbRDR6-qrt-F2 and NbRDR6-qrt-R2.

## Supplementary Material

pcae100_Supp

## Data Availability

Nucleotide sequences of pTRSV1, pTRSV1-16K and pTRSV2-SpCas9-gNbPDS were deposited in DDBJ under accession numbers LC764403, LC764404 and LC764405, respectively. Amplicon sequencing data are available in DDBJ under accession numbers PRJDB17821 and PRJDB17822. Data supporting the findings of this work are available within the paper and its **Supplementary Information files** and from the corresponding author upon reasonable request.
