# Supplementary material for: Heritable Tissue-Culture-Free Gene Editing in Nicotiana benthamiana through Viral Delivery of SpCas9 and sgRNA
Source: Plant Cell Physiol. 2024 Aug 31;65(11):1743–50. doi: 10.1093/pcp/pcae100 (PMC11631083; doi:10.1093/pcp/pcae100)
Supplement: pcae100_Supp [file pcae100_supp.zip › suppl_data/pcp-2024-e-00195-File005.docx]

**
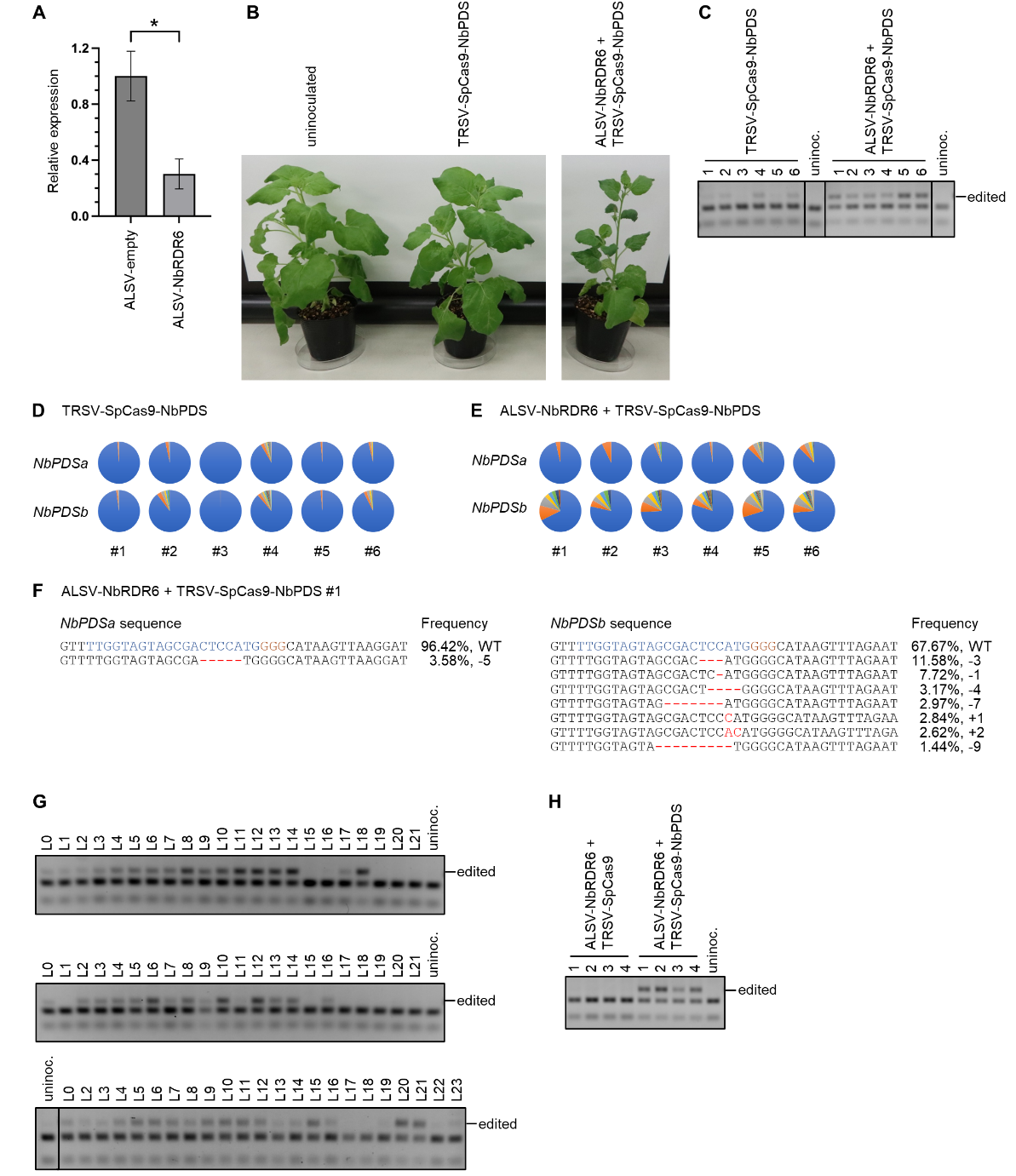
**

**Fig. S1.** **Editing of the *NbPDS* gene by the TRSV-SpCas9-NbPDS vector.** (A) Relative expression analysis of the *NbRDR6* gene in uninoculated upper leaves of *N. benthamiana* plants inoculated with ALSV-empty or ALSV-NbRDR6 by reverse transcription quantitative PCR at 14 days post inoculation (dpi). The *N. benthamiana* *PROTEIN PHOSPHATASE 2A (NbPP2A)* gene was used as an internal control. Mean values and standard deviations of 15 samples from three independent experiments are presented. Statistical significance was determined using two-tailed Student’s *t*-test. * *p* = 2.02×10^-13^. (B) Typical symptoms of plants inoculated with virus vectors denoted at 23 dpi from the TRSV inoculation. (C) CAPS analysis of the tenth upper leaves from the TRSV-inoculated leaves of *N. benthamiana* plants inoculated with TRSV-SpCas9-NbPDS with or without pre-inoculation of ALSV-NbRDR6. Numbers indicate independent plants. Lanes were rearranged. (D,E) Amplicon sequencing analysis of *NbPDSa* and *NbPDSb* in the tenth upper leaves from the TRSV-inoculated leaves of plants inoculated with TRSV-SpCas9-NbPDS (D) and ALSV-NbRDR6 and TRSV-SpCas9-NbPDS (E). The unedited and edited sequences are shown in blue and other colors in the order of read counts, respectively. The numbers correspond to the samples shown in (C). (F) Nucleotide sequences around the target sites of *NbPDSa* and *NbPDSb* of the plant inoculated with ALSV-NbRDR6 and TRSV-SpCas9-NbPDS #1 shown in (C) and (E). The sgRNA target sequence and the PAM sequence are shown in blue and brown, respectively. A red letter and hyphen indicate a nucleotide insertion and deletion, respectively. Mutation types (number of nucleotide indels) are indicated. WT indicates the sequence with no mutations. (G) Detection of targeted mutagenesis in the *NbPDS* gene in an inoculated (L0) and upper uninoculated (1st to 23rd; L1–L23) leaves of plants inoculated with ALSV-NbRDR6 and TRSV-SpCas9-NbPDS at 43 (Upper and Middle) and 59 (Lower) dpi from the TRSV inoculation by CAPS analysis. The results of three independent plants are shown. Lanes of the lower panel were rearranged. (H) Detection of targeted mutagenesis by CAPS analysis in the *NbPDS* gene in the tenth upper leaves from TRSV-inoculated leaves of plants inoculated with TRSV-SpCas9 or TRSV-SpCas9-NbPDS pre-inoculated with ALSV-NbRDR6. The positions of undigested bands are indicated as “edited”. “Uninoc.” indicates an uninoculated negative control plant.


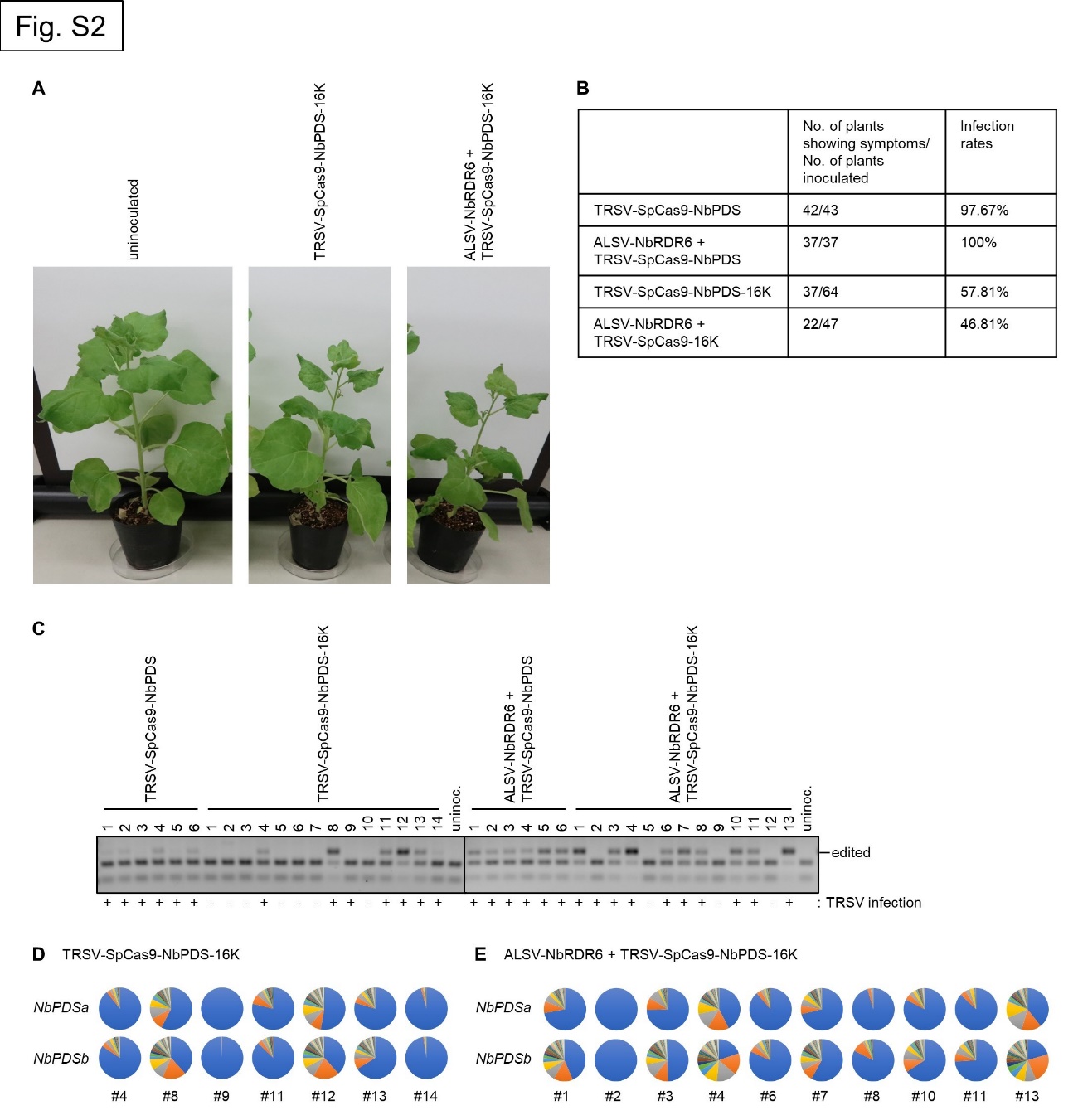


**Fig. S2. Editing of the *NbPDS* gene by the TRSV-SpCas9-NbPDS-16K vector.** (A) Typical symptoms of plants inoculated with virus vectors denoted at 27 dpi from the TRSV inoculation. (B) Infection rates of TRSV-SpCas9-NbPDS and TRSV-SpCas9-NbPDS-16K with or without ALSV-NbRDR6 pre-inoculation determined by the appearance of symptoms. (C) CAPS analysis of the tenth upper leaves from the TRSV-inoculated leaves of *N. benthamiana* plants inoculated with TRSV-SpCas9-NbPDS or TRSV-SpCas9-NbPDS-16K with or without pre-inoculation of ALSV-NbRDR6. Numbers indicate independent plants. For TRSV-SpCas9-NbPDS, ALSV-NbRDR6 + TRSV-SpCas9-NbPDS, and uninoc., the same images as Fig. S1C are shown. The positions of undigested bands are indicated as “edited”. TRSV infection was assessed based on the symptoms. “Uninoc.” indicates an uninoculated negative control plant. (D,E) Amplicon sequencing analysis of *NbPDSa* and *NbPDSb* in the tenth upper leaves from the TRSV-inoculated leaves of plants inoculated with TRSV-SpCas9-NbPDS-16K (D) and ALSV-NbRDR6 and TRSV-SpCas9-NbPDS-16K (E). The unedited and edited sequences are shown in blue and other colors in the order of read counts, respectively. The numbers correspond to the samples shown in (C).

**
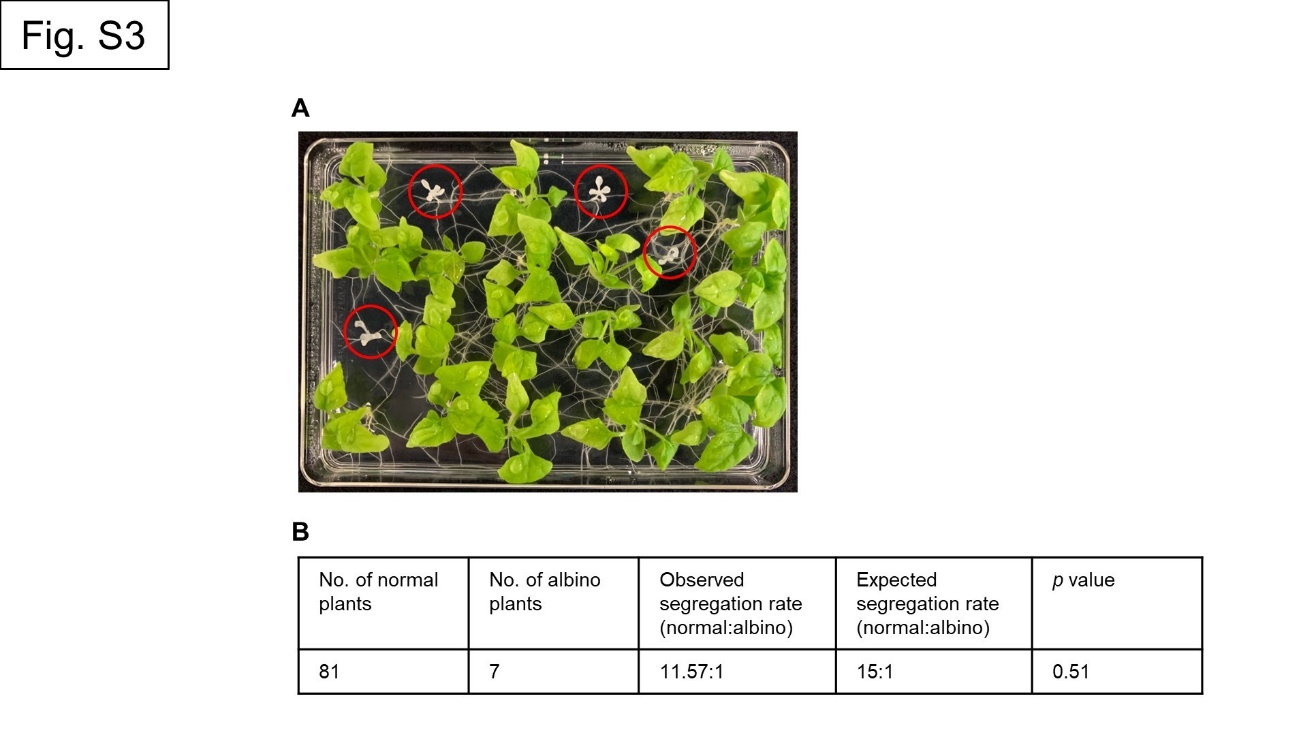
**

**Fig. S3. Phenotype segregation in the offspring of a mutated progeny plant derived from a virus vector-inoculated plant.** (A) Offspring individuals of a mutated progeny that contains a mono-allelic mutation both at *NbPDSa* and *NbPDSb* derived from a plant inoculated with ALSV-NbRDR6 and TRSV-SpCas9-NbPDS. Red circles indicate albino individuals. (B) A ratio of phenotypic segregation. The *p* value was calculated using a chi-squared test.

**
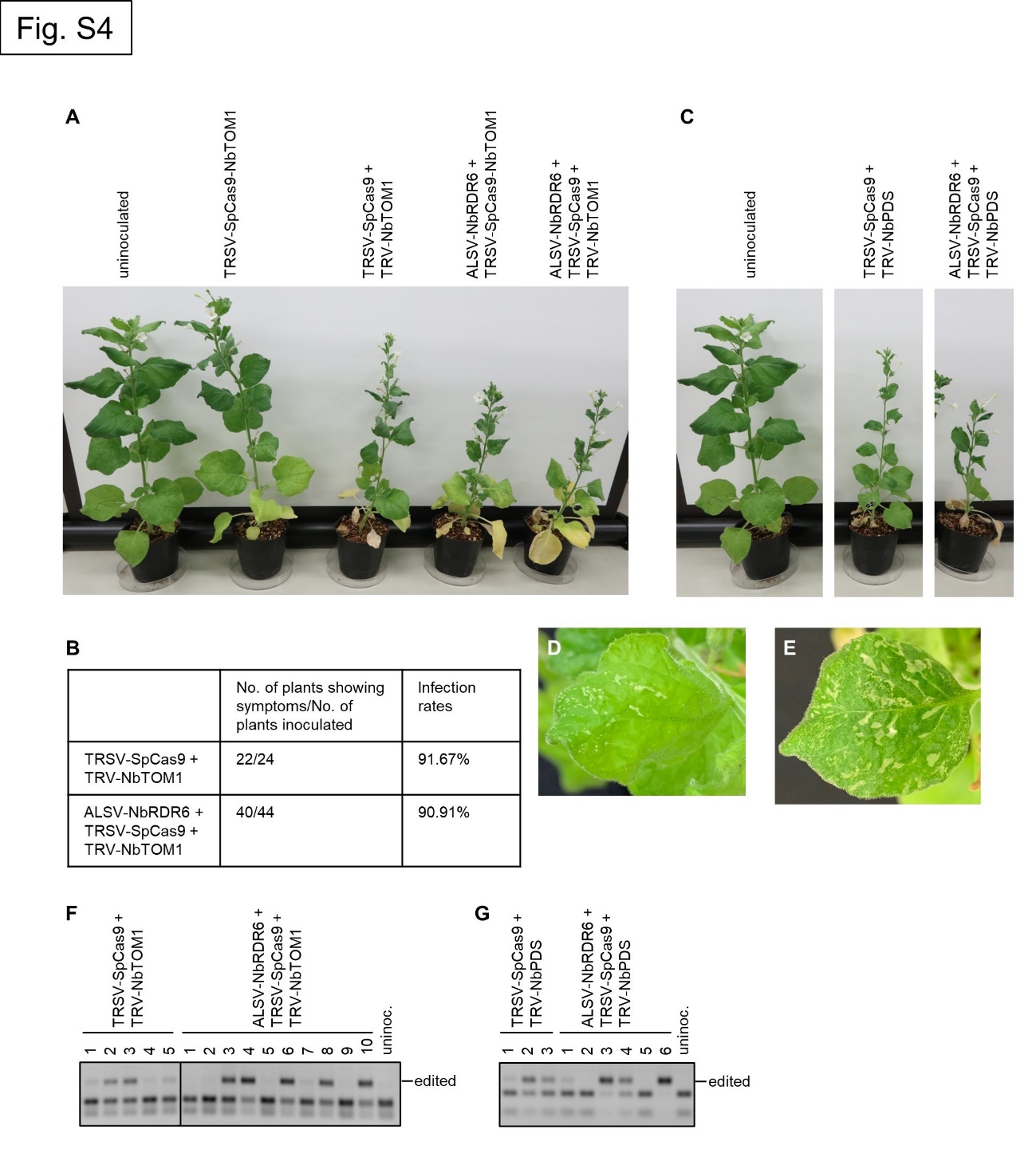
**

**Fig. S4.** **Editing of the *NbTOM1* and *NbPDS* genes by co-inoculation of TRSV-SpCas9 and TRV carrying sgRNA.** (A) Typical symptoms of plants inoculated with denoted virus vectors at 29 dpi from the inoculation of TRSV and TRV. (B) Infection rates of TRSV-SpCas9 and TRV-NbTOM1 with or without ALSV-NbRDR6 pre-inoculation determined by the appearance of symptoms. (C) Typical symptoms of plants inoculated with denoted virus vectors at 29 dpi from the inoculation of TRSV and TRV. (D,E) Examples of partially photobleached upper uninoculated leaves of plants inoculated with TRSV-SpCas9 and TRV-NbPDS (D) or ALSV-NbRDR6, TRSV-SpCas9, and TRV-NbPDS (E). (F,G) Detection of mutations in the *NbTOM1* (F) and *NbPDS* (G) genes by CAPS in the tenth upper leaves from TRSV- and TRV-inoculated leaves of plants inoculated with virus vectors denoted. Lanes were rearranged in (F).

**
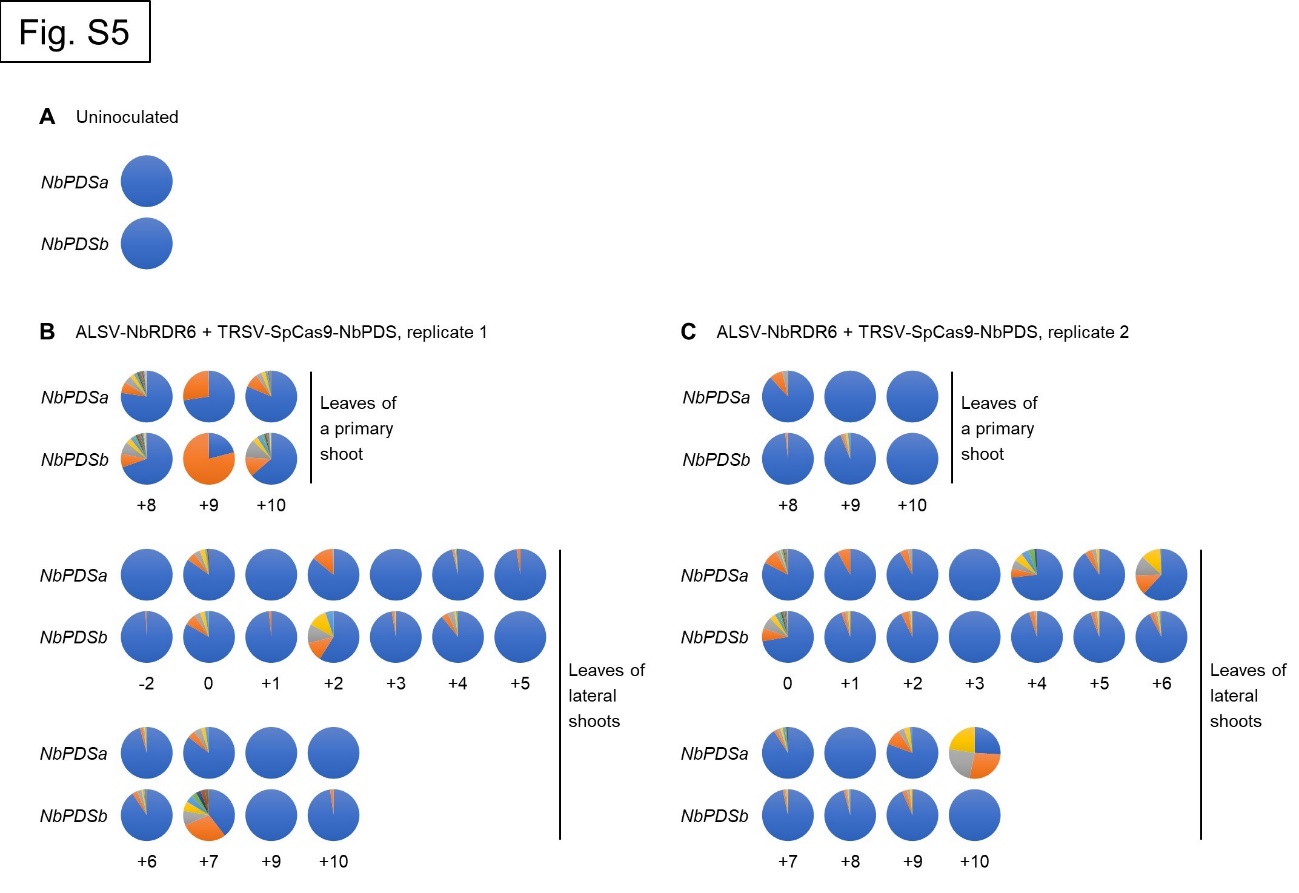
**

**Fig. S5. Distribution of the introduced mutations in *NbPDSa* and *NbPDSb* among leaves from the primary and lateral shoots of plants inoculated with ALSV-NbRDR6 and TRSV-SpCas9-NbPDS.** (A–C) Amplicon sequencing analysis of *NbPDSa* and *NbPDSb* in leaves from an uninoculated plant (A), in leaves from the primary and lateral shoots of plants inoculated with ALSV-NbRDR6 and TRSV-SpCas9-NbPDS replicate 1 (B), and replicate 2 (C). The unedited and edited sequences are shown in blue and other colors in the order of read counts, respectively. Numbers indicate the position of nodes where the lateral branches or leaves arose. Zero, minus, and plus indicate the node of an inoculated leaf, lower nodes, and upper nodes, respectively.


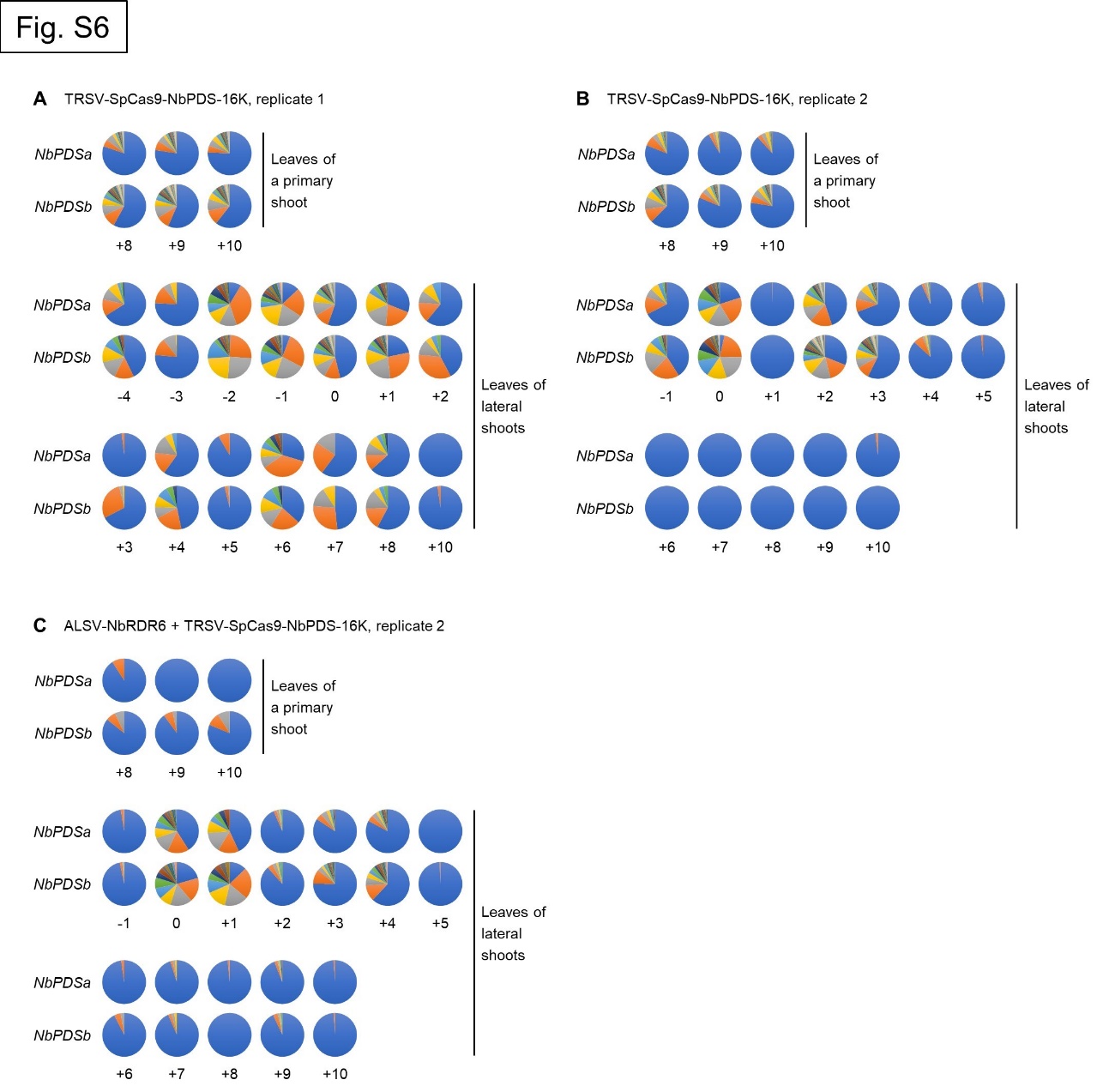


**Fig. S6. Distribution of the introduced mutations in *NbPDSa* and *NbPDSb* among leaves from the primary and lateral shoots of plants inoculated with TRSV-SpCas9-NbPDS-16K with or without ALSV-NbRDR6 pre-inoculation.** (A–C) Amplicon sequencing analysis of *NbPDSa* and *NbPDSb* in leaves from the primary and lateral shoots of plants inoculated with TRSV-SpCas9-NbPDS-16K replicate 1 (A), replicate 2 (B), and in leaves from the primary and lateral shoots of plants inoculated with ALSV-NbRDR6 and TRSV-SpCas9-NbPDS-16K, a replicate of Fig. 3G (C). The unedited and edited sequences are shown in blue and other colors in the order of read counts, respectively. Numbers indicate the position of nodes where the lateral branches or leaves arose. Zero, minus, and plus indicate the node of an inoculated leaf, lower nodes, and upper nodes, respectively.


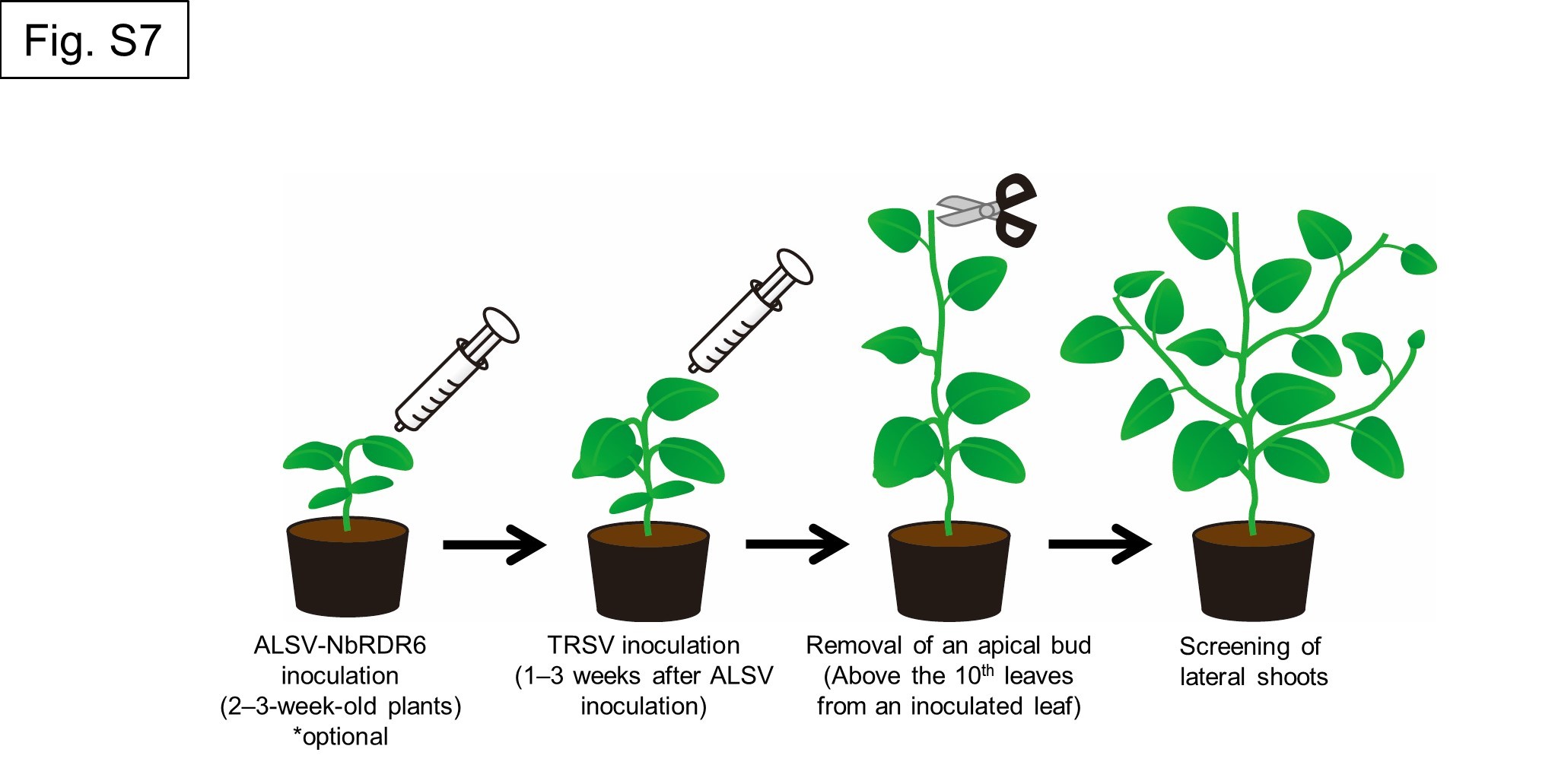


**Fig. S7.** **Scheme of the virus-mediated, heritable, and tissue-culture-free gene editing method developed in this study for an easy isolation of gene-edited plants.**
